# Case Report: Association between cyclic neutropenia and SRP54 deficiency

**DOI:** 10.3389/fimmu.2022.975017

**Published:** 2022-09-08

**Authors:** Melinda Erdős, Oksana Boyarchuk, László Maródi

**Affiliations:** ^1^ Primary Immunodeficiency Clinical Unit and Laboratory, Department of Dermatology, Venereology and Dermatooncology, Semmelweis University, Budapest, Hungary; ^2^ St. Giles Laboratory of Human Genetics of Infectious Diseases, Rockefeller University, New York, NY, United States; ^3^ Department of Children’s Diseases and Pediatric Surgery, I. Horbachevsky Ternopil National Medical University, Ternopil, Ukraine

**Keywords:** signal recognition particle, cyclic neutropenia, granulocyte - colony-stimulating factor (G-CSF), autosomal dominant disease, WES - whole-exome sequencing

## Abstract

Autosomal dominant mutations in the signal recognition particle (SRP) 54 gene were recently described in patients with severe congenital neutropenia (SCN). SRP54 deficiency cause a chronic and profound neutropenia with maturation arrest at the promyelocyte stage, occurring in the first months of life. Nearly all reported patients with SRP54 mutations had neutropenia without a cyclic pattern and showed a poor or no response to granulocyte colony-stimulating factor (G-CSF) therapy. We report here an 11-year-old female patient with cyclic neutropenia and recurrent heterozygous p.T117del (c.349_351del) in-frame deletion mutation in *SRP54*, who showed remarkable therapeutic response to G-CSF treatment. The diagnosis of cyclic pattern of neutropenia was established by acceptable standards. ELANE gene mutation was excluded by using various genetic approaches. The patient described here also had dolichocolon which has not been described before in association with SCN.

## Introduction

Severe congenital neutropenia (SCN) represents a heterogeneous group of genetic disorders characterized by an absolute neutrophil count (ANC) <500 per µL, recurrent, life-threatening bacterial infections and, in some cases, immunological or extra‐hematopoietic abnormalities affecting the pancreas, central nervous system, heart, bone and skin (1–3). Patients with SCN have an extraordinarily high risk for leukemic transformation (4). To date, molecular abnormalities in more than twenty genes have been identified as a cause of SCN (5). The pathways linked to the genetic defects of SCN involve cellular stress mechanisms, like unfold response (*ELANE*) (6, 7), endoplasmic reticulum (ER) stress (*G6PC3*, *JAGN1*) (8, 9), defective endosome trafficking (*VPS13B*, *VPS45*) (10, 11), impaired intracellular glucose homeostasis (*G6PC3*), and defective ribosome biogenesis (*SBDS*, *DNAJC21*, *EFL1*) (12–14). In about 25% of patients with a clinical history suggestive of SCN, the genetic defect remains unknown.

Cyclic neutropenia (CN) is characterized by periodical oscillation of ANC (15), The oscillation cycle of neutrophils is on average 21 days and can be combined with the oscillations of other blood cells including monocytes, lymphocytes and platelets. In the majority of cases CN is associated with mutation in *ELANE* gene, although the mechanism of ANC oscillating is not completely clear (15). There were reports of cases of cyclic pattern of neutropenia in patients with a *HAX1* mutation and biallelic *G6PC3* mutation (16, 17).

Recently, *de novo* dominantly inherited mutations in the signal recognition particle (SRP) 54 genes were described and found to represent the second most common cause of CN with maturation arrest (18). Only one case of cyclic neutropenia associated with *SRP54* mutation has been described (19). SRP54 is an evolutionarily conserved protein which is a key component of the ribonucleoprotein complex mediating the co-translational targeting of secretory and membrane proteins to the ER (18). Patients with SRP54 deficiency typically have chronic and profound neutropenia with maturation arrest at the promyelocyte stage, occurring early in life (18, 19). Bone marrow examination of patients with *SRP54* mutation revealed a major dysgranulopoiesis and features of cellular ER stress and autophagy. Neutropenia may associate with severe neurodevelopmental delay (autistic behavior) and an exocrine pancreatic insufficiency requiring enzyme supplementation. Patients may present with atypical phenotype with normal peripheral neutrophil counts and intermittent granulocyte maturation arrest. A recently published cohort analysis revealed variable immunological and clinical phenotypes in individuals with the same mutation, even in the same family (18). The influence of genetic modifiers in neutrophils may be a possible explanation.

Herein, we report an 11-year-old female patient with cyclic neutropenia and recurrent heterozygous p.T117del (c.349_351del) in-frame deletion in *SRP54*, who presented with dolichocolon and was successfully treated with G-CSF.

## Methods

### Clinical evaluation

The patient and her family members were interviewed, examined, treated and monitored at the Ternopil Regional Children’s Hospital in Ukraine. Medical records were obtained from the electronic registry of the Ternopil University Clinic. The parents of the patient gave written informed consent to conduct the study and for publication of data. All procedures were performed in accordance with the ethical standards of the Institutional Research Committee.

### Blood cells and immunological studies

Blood cell analysis was performed by routine hematological assays. Lymphocyte subsets of peripheral blood mononuclear cells were determined by immunofluorescent staining and flow cytometry. Cell surface markers were detected by using monoclonal antibodies to CD3, CD4, CD8, CD19, CD16, and CD56 cell surface antigens. Serum levels of IgG, IgA, IgM, C3 and C4 were measured by standard immunological assays.

### Whole-exome sequencing *(WES)* and panel sequencing

Genomic DNA from the patient and her parents was isolated with the Gen Elute Blood Genomic DNA kit (Sigma-Aldrich, St. Louis, Missouri, USA)WES. WES was performed at the NY laboratory. At the New York Genome Center and the Rockefeller University an Illumina HiSeq 2500 machine and the Agilent 71 Mb SureSelect exome kit were used, in accordance with the manufacturer’s instructions (20). *Panel sequencing*. A courtesy genetic analysis supported by the Jeffrey Modell Foundation was also performed at an Invitae Laboratory focusing on 407 primary immunodeficiency genes (21). Genomic DNA was enriched for targeted regions using a hybridization-based protocol, and sequenced using Illumina technology. All targeted regions were sequenced with ≥50x depth. Reads were aligned to a reference sequence. Clinically significant observations were confirmed by orthogonal technologies.

### Targeted gene sequencing

Mutational analysis of *ELANE* was performed in the Laboratory of Immunopathology and Genetics at the University of Lodz, Poland. Sequences were analyzed by amplifying exons and flanking intronic regions of *ELANE* by PCR. The PCR primers and sequencing primers are available on request. Amplicons were sequenced with the Big Dye Terminator cycle sequencing kit (Applied Biosystems, Foster City, California, USA) and targeted regions were analyzed by an ABI 3130 capillary sequencer (Applied Biosystems). Sequence variants were determined by using the Sequencer v 5.0 software to identify the position of mutations.

## Results

The 11-year-old female patient was the only child of non-consanguineous parents. She was born from a full-term pregnancy complicated with placental dysfunction, polyhydramnion and pyelonephritis. Her birth weight and length were 3,650 g and 55 cm, respectively. After birth, she developed purulent conjunctivitis which was successfully treated with local antibiotics. She was breastfed for up to 1 year of age and received all vaccines of the Ukrainian mandatory vaccination program, except for hepatitis B vaccine for unknown reasons. Her 27-year-old mother and the 38-year-old father had recurrent herpes labialis and herpetic keratitis, respectively. The family history was negative for dysmorphic features, hematological disease, immune deficiency, or neonatal deaths.

**Figure 1:**
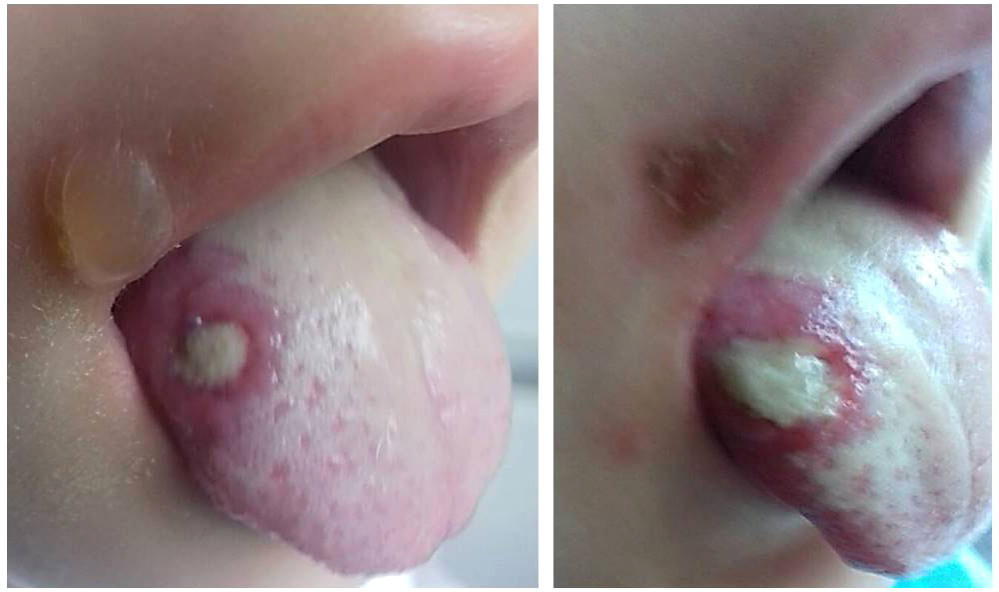
Deep ulcer on the right side of the tongue. Bullous lesion near the edge of the mouth is also visible. The pictures were taken three days apart.

**Figure 2:**
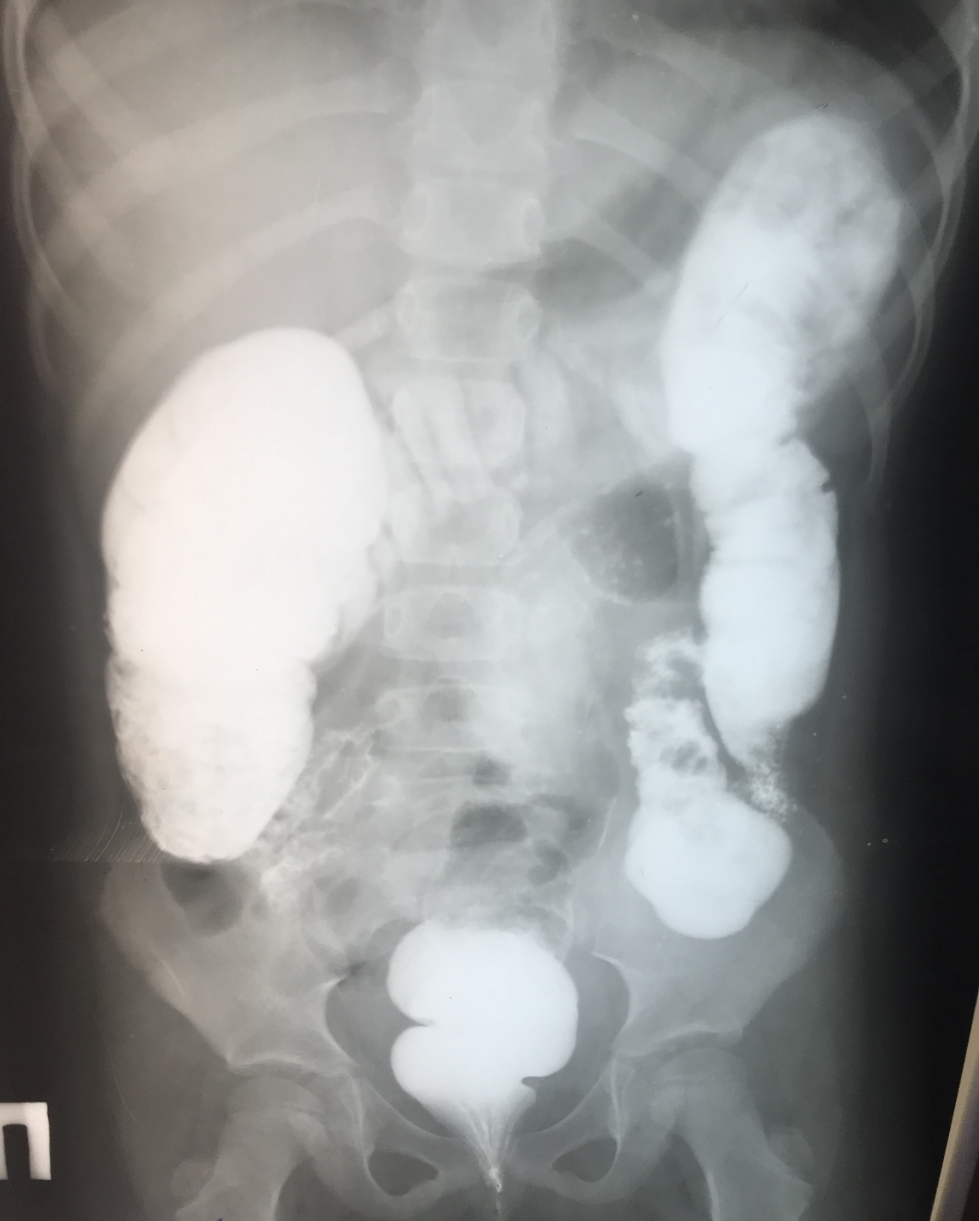
Irrigography by using contrast barium enema revealed dolichocolon at the age of 5 years.

The first disease manifestations at 10 months of age include stomatitis, gingivitis and cervical lymphadenomegaly. During the second year of life she had recurrent stomatitis, impetigo and skin abscesses. At 2 years of age, she was evaluated for anemia, recurrent episodes of fever, mucositis and urinary tract infections. Ulcerative stomatitis recurred in every 2 to 4 weeks with fever, gingivitis, painful cervical lymphadenomegaly and angular cheilitis (**Figure 1**). Later on, exacerbations have been observed in every 3 weeks and lasted for 4-6 days. From the age of 3 years, the patient was treated with chronic constipation and radiology examination revealed dolichocolon (**Figure 2**). Later she developed steatorrhea and constipation with intermittent diarrhea. Abdominal ultrasound revealed an enlarged liver and enlarged, hyperechoic pancreas with homogeneous pancreatic parenchyma. She received pancreatic enzyme preparation (Creon, 30.000 IU/day) with moderate effectiveness. Pancreatic enzyme preparation was given to the patient because the ultrasound examination showed persistent changes in the pancreas, and the long-term constipation alternated periodically with episodes of diarrhea. She did not present with psychomotor developmental delay or autistic behavior, but the parents noted that she was irritable, often nervous and emotionally unstable. Neurological examination did not show any organic abnormalities.

**Figure 3:**
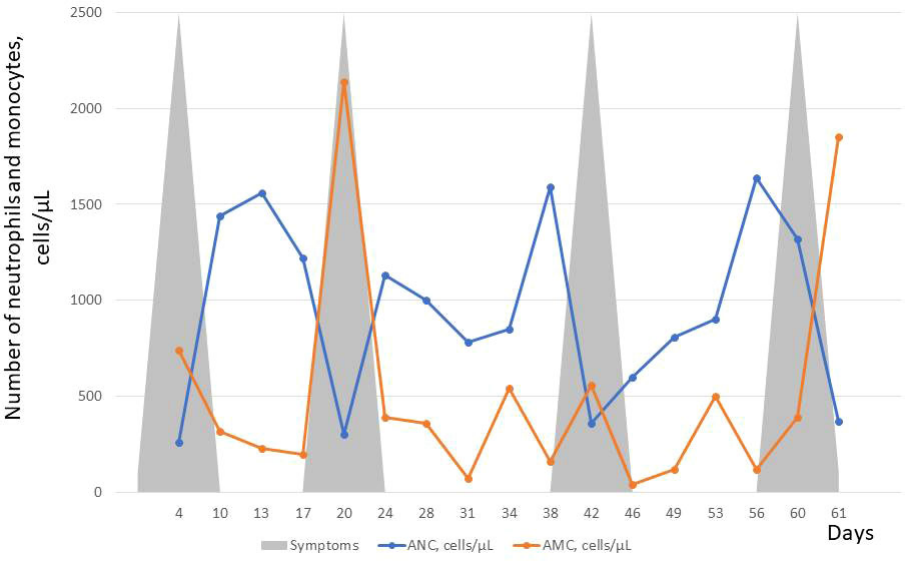
Absolute neutrophil count (ANC) and absolute monocyte count (AMC) over a period of 61 days before starting G-CSF therapy. About 20 day ANC cycles are presented by counting cell number at every 3 or 4 days. In contrast, AMC cycling was not observed over time. Rather, compensatory monocytosis were observed during the 2^nd^ and 4^th^ neutropenia cycles. Upper and lower respiratory tract infections were observed during each neutropenia cycles shown schematically by grey triangles.

Laboratory tests revealed neutropenia with compensatory monocytosis, anemia (lowest RBC: 3.16 T/L, lowest Hgb: 9.4 g/dL) with normal serum iron concentration and normal platelet count. Serial blood counts showed a cyclical pattern of neutropenia occurring in a 20 days period (**Figure 3**). Because of adherence issues we could perform counting of blood cells only in every 3 or four days (**Figure 3**). Serum amylase level was normal and stool analysis revealed normal elastase activity. Bone marrow cytology at 7 years of age revealed normocellularity, with polymorphic composition and no blast infiltration. Bone marrow aspirations also showed signs of a slight dysgranulopoiesis with eosinophia (11.6%) and myeloid maturation arrest at the promyelocyte stage. It was performed in the nadir phase (370 neutrophils/µL). Serum IgG, IgA, IgM, and IgE levels and lymphocyte immunophenotypes were in the normal ranges.

Mutational analysis was first performed to search for possible *ELANE* sequence variant. Direct sequencing of all exons and exon-intron boundaries of *ELANE* NM_001972.3 did not reveal pathogenic variants that could predispose to cyclic neutropenia. Next, WES analysis was performed at the New York Genome Center and the Rockefeller University and revealed a heterozygous p.T117del (c.349_351del) in-frame deletion mutation in the *SRP54* gene, which was previously reported to be causal (18, 19, 22–24). The same sequence variant was found in an Invitae Laboratory by using different sample. None of these studies indicated *ELANE* mutation. Importantly, screening for genetic causes of neutropenia did not reveal mutations in other congenital neutropenia genes (the list of genes we have tested is available on request).

G-CSF treatment was initiated at the age of 10 years at the initial dose of 5 mcg/kg for at least 4 days in every 3 weeks. This treatment regimen resulted in ANC counts above 1.000/µL and reduced the frequency and severity of infections. The patient has remained on G-CSF treatment for the past year without any adverse events. This regimen was sufficient to maintain the patient’s ANC above 1.000/µL. In every two months, mild aphthous ulcers appeared which healed without additional treatment in 1-2 days. Her growth parameters remained in the normal range for ages. The parents also noted that the girl became much calmer, and she had no episodes of behavior change and irritation. Due to concerns about side effects, the parents refused increase of the dose of G-CSF.

## Discussion

SRP54 deficiency is a recently described cause of SCN. Mutations in *SRP54* cause syndromic neutropenia with Shwachman-Diamond syndrome-like features. Patients with SRP54 deficiency show a wide spectrum of immunological and clinical manifestations, ranging from mild asymptomatic neutropenia and febrile illnesses to severe neutropenia and life-threatening infection. Most patients with SCN receive long-term treatment with G-CSF and respond to this treatment. Lifetime treatment with G-CSF is indicated in patients responding to standard doses (5 mcg/kg per day). In those requiring higher doses of G-CSF or those who have transformed into myelodysplasia or acute myeloid leukemia (MDS/AML), hematopoietic stem cell transplantation should be considered, especially if an appropriate HLA-matched donor is available. In a large cohort of 23 patients with SRP54 deficiency, nearly all showed a poor or no response to G-CSF therapy (18). In contrast to ELANE deficiency, no development of AML was observed after a median follow-up for 15 years in this large cohort.

Up to date, 30 cases of patients with SRP54 deficiency were reported in the medical literature and all but one patient (19) had isolated neutropenia without a cyclic pattern (18, 19, 22–24). We report here the second patient with the p.T117del *SRP54* mutation who developed cyclic neutropenia showing cycles of approximately 20-days interval (**Figure 3**). The patient described by Carapito et al. was 8 years old, when he was started on G-CSF at 5 mcg/kg every other day, with improvement of neutrophil counts, mucositis, and infections (18). He continued to do well on G-CSF therapy and was 18 years of age, when his case was published. Like this patient, our patient showed good therapeutic response to G-CSF. We are not aware of more published data on remarkable therapeutic efficacy of G-CSF in patients with SRP54 deficiency but unpublished observation may exist. Currently, there is not convincing evidence for relationship between phenotype (cyclic pattern) of SRP54 deficiency and good response to G-CSF. Further studies and observations of more cases are needed for confirmation of such relationship.

**Figure 4:**
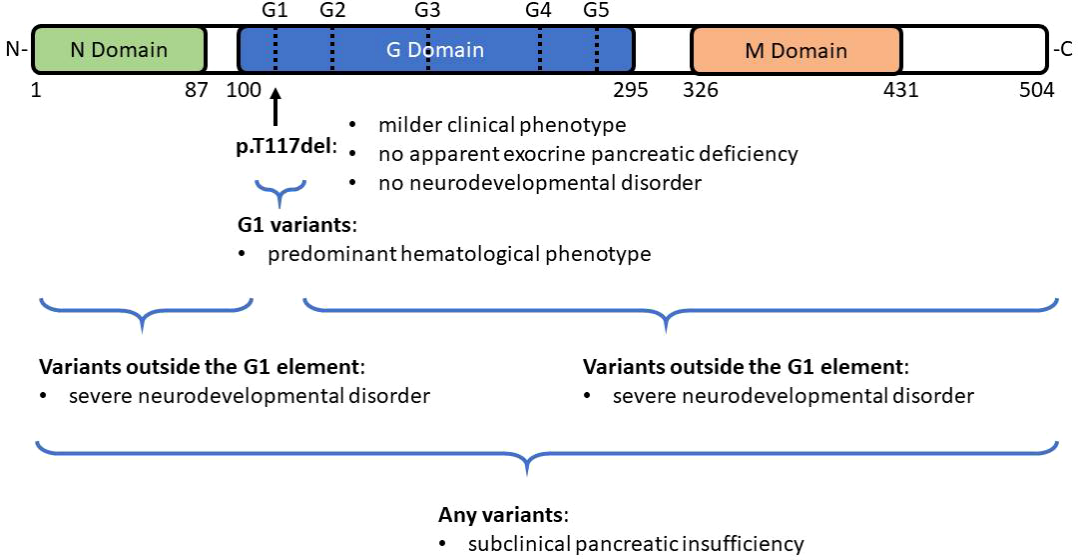
Domain structure of SRP54. SRP54 has three functional domains: N-terminal domain (N-domain), central GTPase domain (G domain), and C-terminal domain (M domain). All the mutated residues in *SRP54* are located around the G domain which contains five specific G elements (G1-G5). G1 variants, like the recurrent p.T117del mutation have been associated with a predominant hematological phenotype. In contrast, patients with G variants that reside outside the G1 element present with a severe neurodevelopmental disorder and in some cases with exocrine pancreatic deficiency. Subclinical pancreatic insufficiency appears widespread throughout the different variants.

Previous studies also suggest genotype-phenotype relationships (18, 19). SRP54 has three functional domains: N-terminal domain (N-domain), central GTPase domain (G domain), and C-terminal domain (M domain) (**Figure 4**). All the mutated residues in *SRP54* are located around the G domain which contains five specific G elements (G1-G5). G1 variants, like the p.T117del mutation, have been associated with a predominant hematological phenotype (**Figure 4**). However, subclinical pancreatic insufficiency appears widespread throughout the different variants. Further, patients with G variants residing outside the G1 element present with a severe neurodevelopmental disorder (extreme delayed speech, intellectual disability) and in some cases with exocrine pancreatic deficiency (18, 19). Published patients with p.T117del mutation were also observed as having a milder clinical phenotype with milder neutropenia both in quantitative terms and with respect to the age of first clinical manifestations with no apparent exocrine pancreatic deficiency or neurodevelopmental disorder. In the study of Bellanné-Chantelot et al, only 2 from 18 patients with *SRP54* mutations interacting directly with the G1 element had an extra-hematological phenotype including moderate exocrine pancreatic insufficiency in one case and severe intellectual disability with autistic traits in another (18). In the latter case the patient had a familial history of severe neurological disorders without known neutropenia, so it could not be excluded that his neurodevelopmental delay is a result of another cause. In contrast, our patient with the p.T117del mutation presented with gastrointestinal manifestations including steatorrhea, constipation and intermittent diarrhea. The association of dolichocolon with a SRP54 mutation is intriguing and the possible causal relationship remains to be elucidated. The patient presented here had hyperirritability and emotional instability but no neurological abnormalities were found. Patients with SCN, especially those with the ELANE mutation may often develop periodontitis (25). This dental anomaly was not observed in our patient and PubMed search did not reveal an association of SRP54 mutation with the development of periodontitis suggesting that this genetic form of SCN may be clinically milder than neutrophil elastase gene defect.

**Figure 5:**
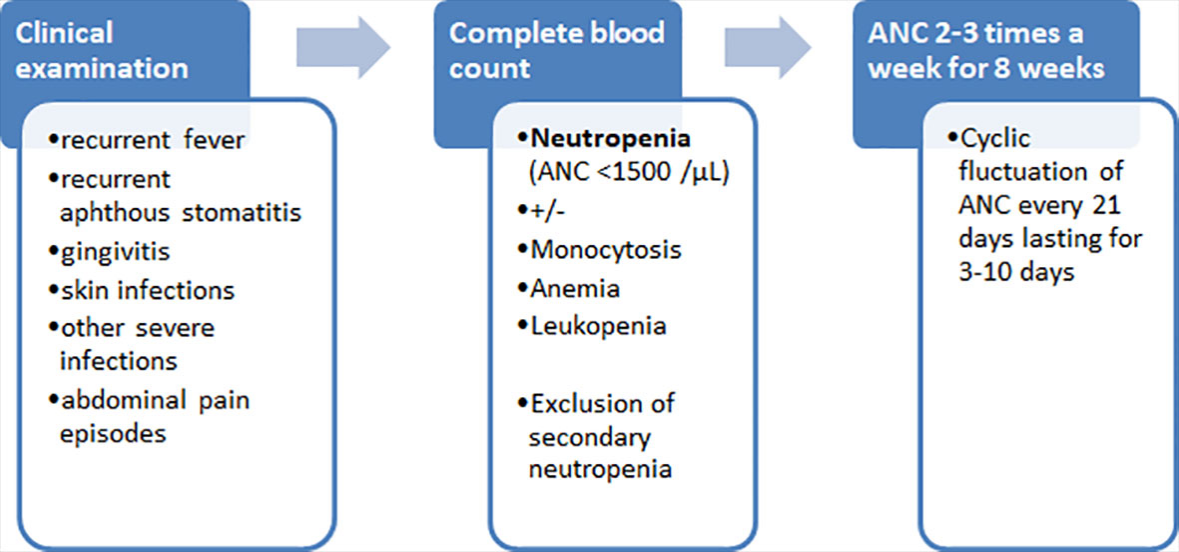
Diagnostic algorithm of cyclic neutropenia.

In summary, we present here a patient with cyclic neutropenia associated with heterozygous p.T117del (c.349_351del) in-frame deletion mutation in *SRP54*. Cyclic neutropenia is a rare hematological condition considered as an autosomal dominant disease caused primarily by *ELANE* gene mutations and characterized by regular fluctuations in blood neutrophil counts, leading to periodic neutropenia. Although in nearly all patients with SRP54 deficiency neutropenia present without a cyclic pattern, our case and the previously reported patient with p.T117del mutation suggest that SRP54 deficiency should also be considered as a possible genetic cause of cyclic neutropenia. We provided here data on successful treatment of an SRP54 deficient patient by administration of G-CSF, in contrast to nearly all previously reported patients with *SRP54* mutations who presented with a poor or no response to G-CSF therapy. Finally, for the general readers, we provide our proposed algorithm of to help the diagnosis of cyclic neutropenia (**Figure 5**).

## Data availability statement

The original contributions presented in the study are included in the article/supplementary material. Further inquiries can be directed to the corresponding author.

## Ethics statement

The studies involving human participants were reviewed and approved by Ternopil University. Written informed consent to participate in this study was provided by the participants’ legal guardian/next of kin.

## Author contributions

ME: performing bioinformatics analysis and writing the initial draft. OB: conducting clinical research and patient care, editing the initial draft. LM: formulation of research goals, writing the final draft. All authors contributed to the article and approved the submitted version.

## Funding

This study was supported by the J Project physician education and clinical research collaboration program and the Foundation for Children with Immunodeficiencies.

## Acknowledgments

The authors thank K Babol-Pokora (Laboratory of Immunopathology and Genetics, Central Clinical Hospital, Medical University of Lodz) for *ELANE *gene sequencing. We thank B Boisson and J-L Casanova for helpful discussion and allowing their facilities for WES analysis.

## Conflict of interest

The authors declare that the research was conducted in the absence of any commercial or financial relationships that could be construed as a potential conflict of interest.

## Publisher’s note

All claims expressed in this article are solely those of the authors and do not necessarily represent those of their affiliated organizations, or those of the publisher, the editors and the reviewers. Any product that may be evaluated in this article, or claim that may be made by its manufacturer, is not guaranteed or endorsed by the publisher.
